# Leaf ^13^C and ^15^N composition shedding light on easing drought stress through partial K substitution by Na in eucalyptus species

**DOI:** 10.1038/s41598-021-99710-1

**Published:** 2021-10-11

**Authors:** Nikolas Souza Mateus, Antonio Leite Florentino, Jessica Bezerra Oliveira, Elcio Ferreira Santos, Salete Aparecida Gaziola, Monica Lanzoni Rossi, Francisco Scaglia Linhares, José Albertino Bendassolli, Ricardo Antunes Azevedo, José Lavres

**Affiliations:** 1 Center for Nuclear Energy in Agriculture, University of São Paulo, Avenida Centenario, 303. CP 96, Piracicaba, CEP 13416-000 Brazil; 2 College of Agriculture Luiz de Queiroz, University of São Paulo, Piracicaba, 13418-900 Brazil

**Keywords:** Photosynthesis, Plant physiology, Stomata, Drought

## Abstract

This work aimed to investigate the partial K-replacement by Na supply to alleviate drought-induced stress in *Eucalyptus* species*.* Plant growth, leaf gas exchange parameters, water relations, oxidative stress (H_2_O_2_ and MDA content), chlorophyll concentration, carbon (C) and nitrogen (N) isotopic leaf composition (δ^13^C and δ^15^N) were analyzed. Drought tolerant *E. urophylla* and *E. camaldulensis* showed positive responses to the partial K substitution by Na, with similar dry mass yields, stomatal density and total stomatal pore area relative to the well K-supplied plants under both water conditions, suggesting that 50% of the K requirements is pressing for physiological functions that is poorly substituted by Na. Furthermore, *E. urophylla* and *E. camaldulensis* up-regulated leaf gas exchanges, leading to enhanced long-term water use efficiency (WUE_L_). Moreover, the partial K substitution by Na had no effects on plants H_2_O_2_, MDA, δ^13^C and δ^15^N, confirming that Na, to a certain extent, can effectively replace K in plants metabolism. Otherwise, the drought-sensitive *E. saligna* species was negatively affected by partial K replacement by Na, decreasing plants dry mass, even with up-regulated leaf gas exchange parameters. The exclusive Na-supplied plants showed K-deficient symptoms and lower growth, WUE_L_, and δ^13^C, besides higher Na accumulation, δ^15^N, H_2_O_2_ and MDA content.

## Introduction

The *Eucalyptus* genus is frequently cultivated in regions with low precipitation, inducing plants to drought stress, which is considered the main environmental factor limiting plant development^1^. A range of important adaptive mechanisms of drought tolerance, intended to limit water loss, are well documented. Such adaptations include reduction of the leaf area; biochemical strategies like as osmotic changes to maintain cell turgor; physiological adjustments, such as fast stomatal closure followed by stomatal conductance (*g*_*s*_) reduction and, hence, a drop in transpiration rate (*E*)^2^. Furthermore, tolerant genotypes alleviate stress by lowering their osmotic potential and adjusting their cell elasticity^3^. Along with drought stress, potassium (K) availability is an important issue to *Eucalyptus* development and it has been broadly influencing photosynthetic attributes and promoting environmental stress tolerance^4^. Potassium is known for its contribution as an osmoticum, playing an essential role in plant metabolism, maintaining cell growth and turgor, protein synthesis, enzymatic activation, stomata adjustments, electron transport rate through photosystem II, and nitrogen (N) assimilation^5,6^. In this context, an adequate nutrient management can reduce drought stress impacts by increasing plant water use efficiency (WUE)^7^.

Sodium (Na) is beneficial for plants at low concentrations^8^, replacing K to a certain degree mainly by reducing K requirements^9^. However, the life cycle of non-halophytes (found only in non-saline soils) is inhibited by Na concentrations above 100 mM of NaCl^2^. The Na supplementation seems to enhance leaf gas exchanges^10^, reduce reactive oxygen species (ROS)^11^ and photoinhibition in K-deficient plants^12^. Despite the well-known positive effects of Na supply when K is limiting^4,7,10,12–14^, the potential role of Na in the stomatal movement is not well determined^15^ and may offer a way to improve plants’ leaf gas exchange parameters. Nonetheless, since solutes accumulation reduce leaf osmotic potential^9^, Na supply may also enhance drought tolerance by maintaining water uptake during drought periods^7^ and improving plant’s water balance^16^. However, the physiological mechanisms underlying a partial K-replacement by Na in *Eucalyptus* under drought conditions remain unclear, being extremely important to enhance management practices in order to face future climate changes. Moreover, due to the well-known importance of K fertilizer for crop yield, potash consumption has increased dramatically in most regions of the world^17^, justifying the great interest in understanding K and Na dualism for the improvement of plant yield with cheaper fertilizers, as NaCl. Positive effects in growth stimulation due to Na supply in place of the more expensive K fertilizer were observed both in natrophilic and glycophytic plant species grown under (i) soil with low concentration of available K or Na, (ii) highly K-fixing soils and (iii) in irregular rainfall areas^2^. According to the authors, despite the genotypic differences, a huge amount of the expensive K supply would be necessary to stimulate plant growth in these situations, supporting the fertilizer strategy with NaCl.

The influence of K on CO_2_ assimilation (*A*) is major linked to mesophyll diffusion conductance^18^, while changes in environmental conditions such as drought stress, affect greatly *g*_*s*_, and therefore the internal to ambient CO_2_ concentration ratio (*C*_*i*_/*C*_*a*_)^19^, leading to alterations in photosynthetic discrimination in C_3_ leaves. The carbon (C) isotope fractionation method (δ^13^C) can be divided in two main discriminating steps during CO_2_ fixation in C_3_ plants: 1) discrimination during CO_2_ diffusion from the ambient air into the leaves through the stomata and 2) discrimination by the RuBisCO carboxylation, which preferentially uses ^12^CO_2_ relative to ^13^CO_2_^20^. By contrast the N isotope composition (δ^15^N) is a good predictor of the plant growth status and N metabolism^21^. Plants with higher photosynthetic capacity and transpiration efficiency accumulate more N, the most growth-limiting nutrient element for plants. In this context, the δ^13^C and δ^15^N have been used to gain insights into the effect of growing conditions on C and N metabolism, as also the mechanisms responsible for plants responses to nutritional status and abiotic stress throughout its whole cycle, since WUE, N acquisition and C uptake may be correlated^22–25^. However, studies regarding the interactive effect of partial K substitution by Na on the δ^13^C and δ^15^N of the *Eucalyptus* seedlings grown under drought condition are scarce.

Despite the similarities between the Na^+^ and K^+^ hydrated ionic radii, the extent of K replacement by Na in plant nutrition not only varies among species, but can also be harmful. Most of agriculturally crops are intolerant to salt, being inhibited by high Na concentrations^15^. Under salinity conditions, which affect more than 20% of the global agriculture lands, the Na exceeds the cells’ ability to compartmentalize Na^+^ in the vacuole, increase the osmotic potential and disrupt cell membrane integrity^21^. As a result, Na accumulates in the cytoplasm, leading to enzyme activity inhibition^26^, and sudden death of entire shoots^27^. Currently, studies regarding Na in plants mainly focus on salinity conditions, i.e., > 100 mM of NaCl, and despite recent advances in understanding drought stress and K supply, little attention has been paid to the combined use of K and Na as an agricultural practice to optimize productivity and efficiency of resources in *Eucalyptus* under drought conditions. Therefore, the effect of partial K-replacement by Na on leaf ^13^C, ^15^N compositions and photosynthesis-related parameters remains unclear, especially in plants with different drought tolerances when subjected to soil–water availabilities. We hypothesized that (i) the partial K substitution by Na has the potential to alleviate the drought-induced stress in *Eucalyptus* by up-regulating growth, physiological and biochemical parameters and (ii) confirm the use of natural isotopic abundance (δ^13^C and δ^15^N) as a reliable indicator of the positive effects of partial K substitution by Na on C and N metabolism throughout plants metabolism cycle. Thus, we applied a multitiered approach at a range of leaf and plant levels to (i) characterize leaf C and N isotope compositions, leaf water potentials, oxidative stress; (ii) quantify the long-term plants water use efficiency; and (iii) evaluate K- and Na-uptake as well as eucalyptus species growth.

## Results

The relative effects (increase or decrease) of partial K replacement by Na (50/50% of K/Na) and exclusive Na-supply (0/100% of K/Na) compared to the well K-supply (100/0% of K/Na) in *E. saligna*, *E. urophylla* and *E*. *camaldulensis* grown under W+ and W− condition can be observed in Supplementary Table 1S.

### Growth parameters

**Figure 1:**
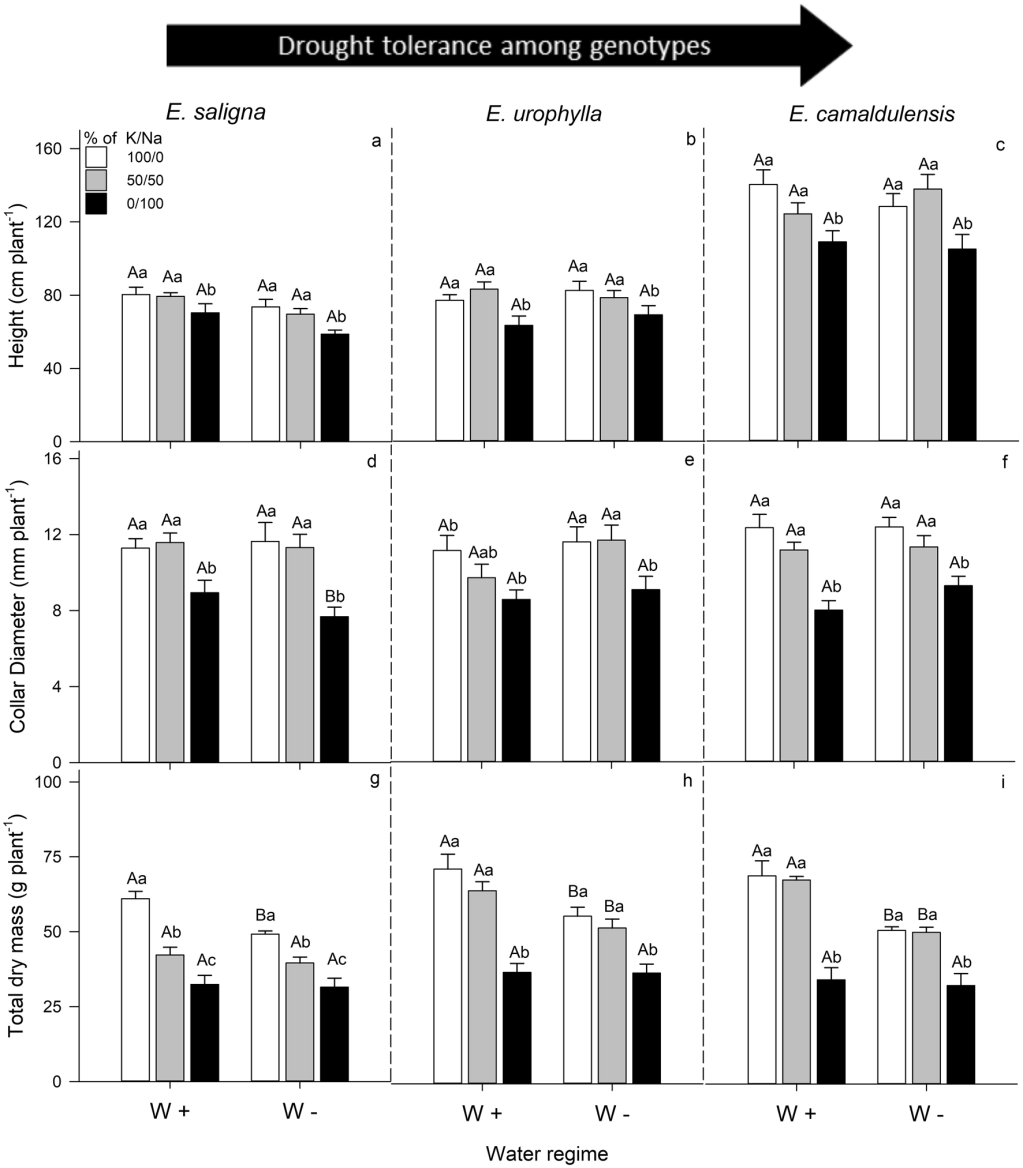
Height (**a**–**c**), collar diameter (**d**–**f**) and total dry mass (**g**–**i**) of *E*. *saligna*, *E*. *urophylla* and *E*. *camaldulensis* seedlings under three soil levels of K/Na in well-watered (W+) and water-stressed (W−) condition. Different uppercase letters indicate difference between water conditions and different lowercase letters indicate differences between the treatments (% of K/Na). Vertical bars indicate standard errors between blocks (n = 4).

A significant decrease was observed in the growth parameters of plants grown under 0/100% of K/Na (exclusive Na-supplied plants) relative to 100/0% of K/Na (well K-supplied plants) or 50/50% of K/Na (partial K replacement by Na) (Fig. 1).

Regardless plant species and water condition, the exclusive Na-supply decreased the height (20%), collar diameter (30%) and total dry mass (TDM) (45%) when compared to well K-supplied plants. Moreover, the partial K replacement by Na decreased the TDM of *E*. *saligna* (up to 30%) compared to well K-supplied plants under both water conditions, while no effects was observed in the drought tolerant species, such as *E*. *urophylla* and *E*. *camaldulensis* (Fig. 1g–i).

Drought had no effect on height and collar diameter, while decreased the TDM of *E*. *saligna* (20%) under well K-supply, as also of *E*. *urophylla* (20%) and *E*. *camaldulensis* (25%) under well K-supply and partial K replacement by Na.

### Leaf gas exchange

**Figure 2:**
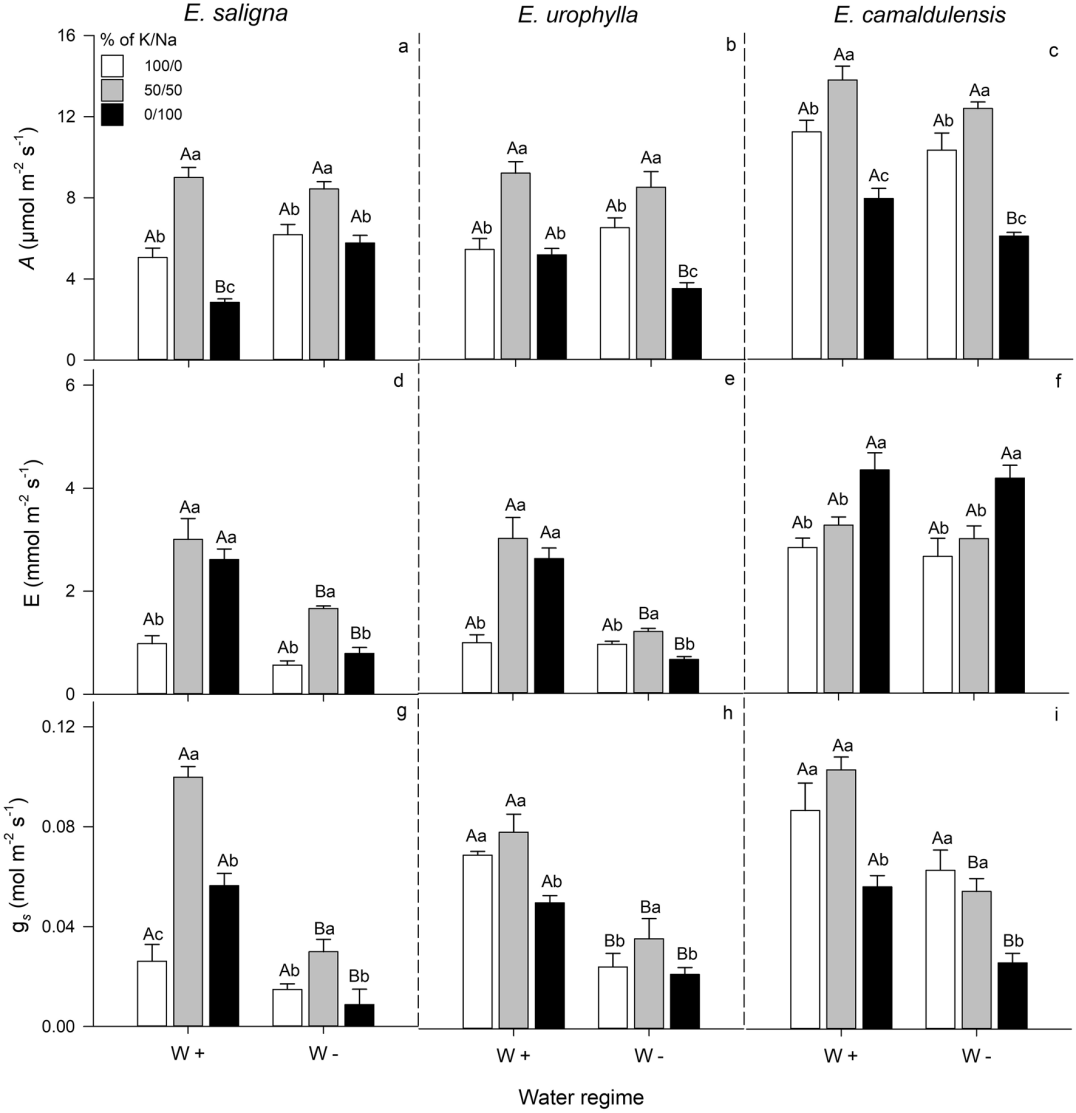
Photosynthetic rate-*A* (**a**–**c**), leaf transpiration rate-*E* (d-f) and stomatal conductance-*g*_*s*_ (**g**–**i**), in leaves of *E*. *saligna*, *E*. *urophylla* and *E*. *camaldulensis* seedlings under three soil levels of K/Na in well-watered (W+) and water-stressed (W−) condition. Different uppercase letters indicate difference between water conditions and different lowercase letters indicate differences between % of K replacement by Na. Vertical bars indicate standard errors between blocks (n = 4).

Overall, the CO_2_ assimilation (*A*), transpiration rate (*E*) and stomatal conductance (*g*_*s*_) were significantly improved by the partial K replacement by Na (Fig. 2).

Regardless water condition, the partial K replacement by Na increased the *A* in all plant species by 45%, while the exclusive Na-supply decreased its values by 40%, relative to well K-supplied plants. Drought-induced stress increased the *A* of *E*. *saligna* (100%), while decreased of *E*. *urophylla* (35%) and *E*. *camaldulensis* (25%) grown under exclusive Na-supply (Fig. 2a–c and Supplementary Table 1S).

The *E* was higher in *E*. *saligna* and *E*. *urophylla* (200%) with partial K replacement by Na compared to well K-supplied plants, while the exclusive Na-supply increased its values only in *E*. *camaldulensis* (55%). The drought decreased the *E* of *E*. *saligna* (57%) and *E*. *urophylla* (67%) grown both under partial K replacement by Na and exclusive Na-supply, compared to plants grown in W+ condition (Fig. 2d–f).

The partial K replacement by Na also increased the *g*_*s*_ of *E*. *saligna* (190%) and *E*. *urophylla* under W− (45%), relative to well K-supplied plants in the same water condition. Moreover, the exclusive Na-supply increased the *g*_*s*_ of *E*. *saligna* under W+, while decreased the *g*_*s*_ of *E*. *urophylla* under W+ and *E*. *camaldulensis* under both water condition, compared to 100/0% of K/Na treatment. The drought also decreased the *g*_*s*_ of *E*. *saligna* and *E*. *camaldulensis* grown under partial K replacement by Na and exclusive Na-supply, and of *E*. *urophylla* under the three soil levels of K/Na (Fig. 2g–i).

### Water use efficiency (WUE_I_, WUE_T_, WUE_L_)

**Figure 3:**
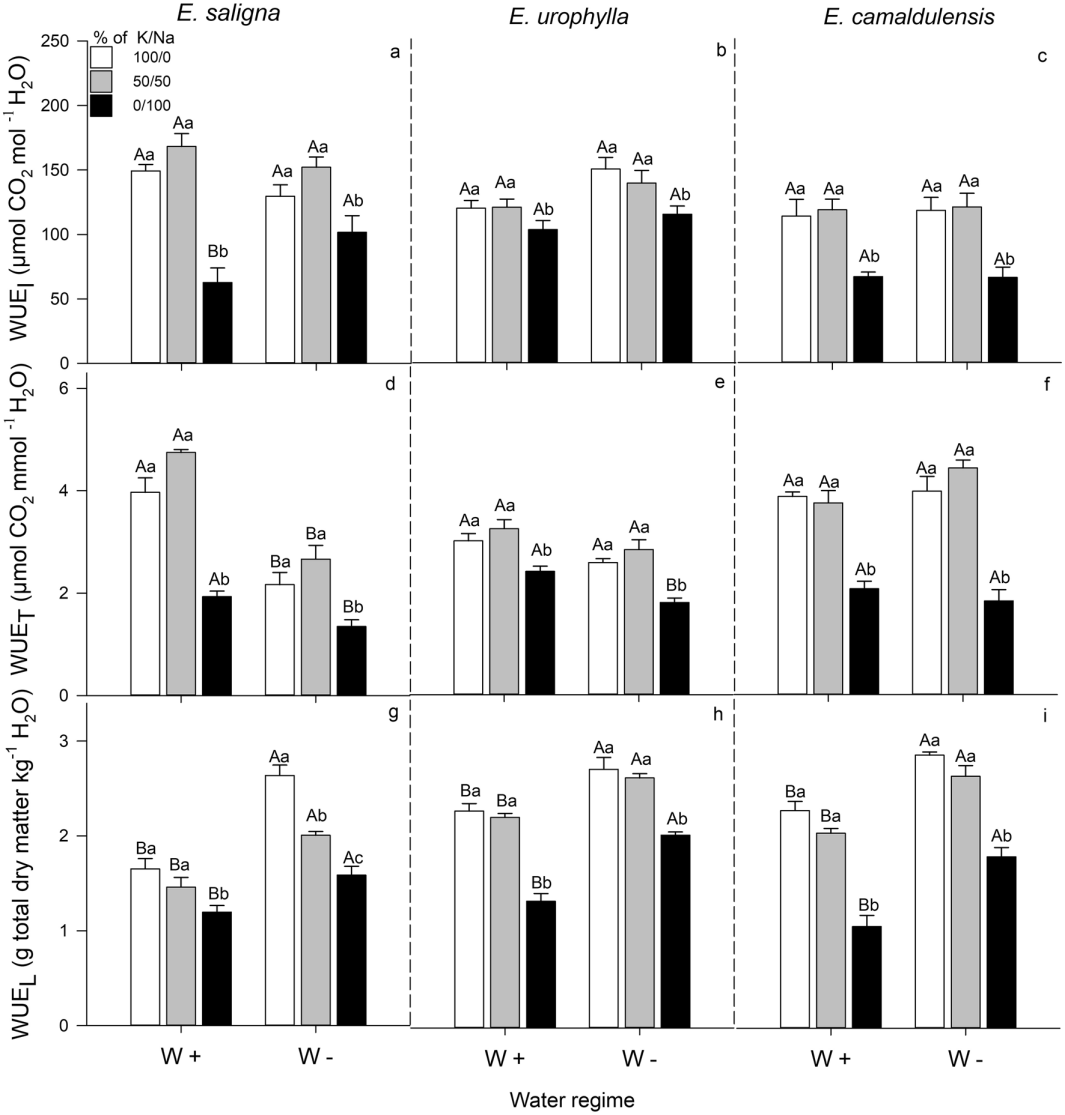
Intrinsic (**a**–**c**), instantaneous (**d**–**f**) and long-term (**g**–**i**) water use efficiencies in leaves of *E*. *saligna*, *E*. *urophylla* and *E*. *camaldulensis* seedlings under three soil levels of K/Na in well-watered (W+) and water-stressed (W−) condition. Different uppercase letters indicate difference between water conditions and different lowercase letters indicate differences between % of K replacement by Na. Vertical bars indicate standard errors between blocks (n = 4).

In general, a significant decrease was observed in WUE of exclusive Na-supplied plants when compared to well K-supplied eucalyptus plants or under partial K replacement by Na, which did not differ from each other, except in the WUE_L_ of *E*. *saligna* subjected to W−condition (Fig. 3).

Regardless water condition, the exclusive Na-supply dramatically decreased the WUE_I_, WUE_T_ and WUE_L_ of *E*. *saligna*, *E*. *urophylla* and *E*. *camaldulensis*, while the partial K replacement by Na also decreased the WUE_L_ of *E*. *saligna* (W−: 25%), relative to well K-supplied plants.

The drought effects on WUE varied among the measurement methods (leaf level or plant scale). Under W− condition, the WUE_I_ was higher in *E*. *saligna* (65%) under exclusive Na-supply, whereas the WUE_T_ decreased in *E*. *saligna* (45%) under the three levels of K/Na rates and in *E*. *urophylla* (15%) at exclusive Na-supply, relative to W+ condition. Moreover, the WUE_L_ was also higher in *E*. *saligna*, *E*. *urophylla* and *E*. *camaldulensis*, under well K-supply, partial K replacement by Na and exclusive Na-supply (Fig. 3 and Supplementary Table 1S).

### Stable isotope natural abundances (δ^13^C, δ^15^N and C/N ratio)

**Figure 4:**
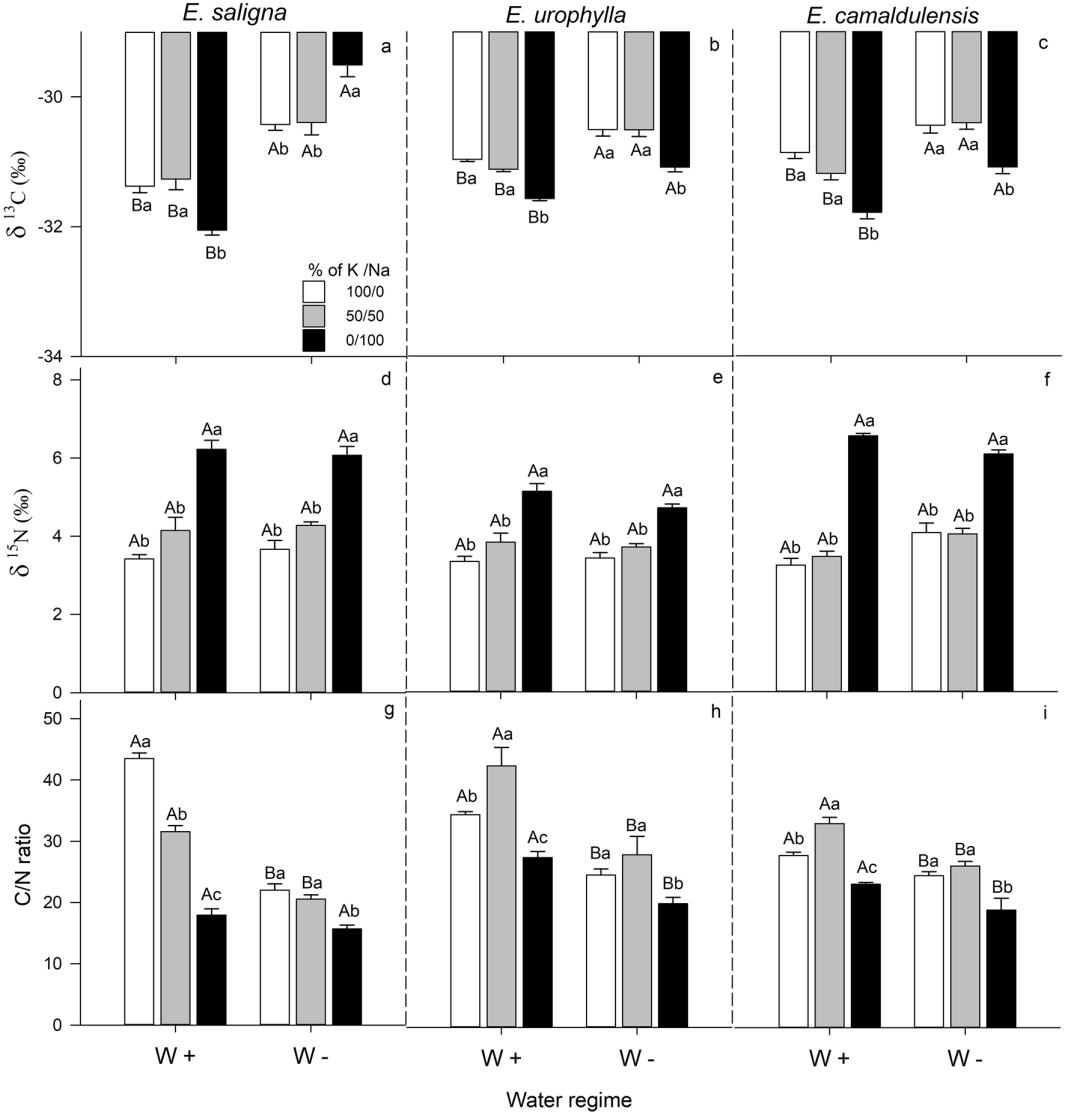
Leaf carbon δ^13^C (**a**–**c**), and nitrogen (δ^15^N) isotope composition (**d**–**f**) and C/N ratio (**g**–**i**) of *E*. *saligna*, *E*. *urophylla* and *E*. *camaldulensis* seedlings under three soil levels of K/Na in well-watered (W+) and water-stressed (W−) condition. Different uppercase letters indicate difference between water conditions and different lowercase letters indicate differences between % of K replacement by Na. Vertical bars indicate standard errors between blocks (n = 4).

Regardless of the water condition, exclusive Na-supplied plants showed the lowest results to δ^13^C (except *E. saligna* under W −) and C/N ratio, as well as the highest results to δ^15^N (Fig. 4). Moreover, plants grown under W −  condition showed the highest δ^13^C and the lowest C/N ratio in all species.

The exclusive Na-supply significantly decreased the δ^13^C to the lowest values in *E*. *saligna* under W+ condition (− 32.0 ± 0.3‰), and for *E. urophylla* and *E. camaldulensis* in both water regimes. In contrast, the values of δ^13^C% for *E*. *saligna* under W− condition were the highest at 0/100% of K/Na (− 29.5 ± 0.3‰). Drought stress increased the mean δ^13^C values of *E*. *saligna* (3%), *E. urophylla* (1.5%) and *E. camaldulensis* (2.3%) under the three soil levels of K/Na, compared to W+ condition (Fig. 4a–c and Supplementary Table 1S).

The highest ^15^N was found in the exclusive Na-supplied plants of *E*. *saligna*, *E. urophylla* and *E. camaldulensis* under both water conditions. The drought had no effects on plants^15^N values (Fig. 4d–f).

Under W+ condition, the exclusive Na-supply decreased the C/N ratio of *E*. *saligna* (60%), *E*. *urophylla* (20%) and *E. camaldulensis* (15%), while the partial K replacement by Na decreased its values for *E*. *saligna* (27%), and increased for *E*. *urophylla* and *E. camaldulensis* by 20%, relative to well K-supplied plants. Moreover, under W− condition, the exclusive Na-supply decreased the C/N ratio of *E*. *saligna* (30%), *E*. *urophylla* (20%) and *E. camaldulensis* (20%), relative to well K-supplied plants at the same water condition. Drought stress decreased the C/N ratio of *E*. *saligna* (40%) at the treatment of well K-supplied plants and partial K replacement by Na, as also of *E*. *urophylla* (30%) and *E. camaldulensis* (15%) at the three soil levels of K/Na, compared to W+ condition (Fig. 4g–i).

### Soluble proteins, H_2_O_2_ and MDA

**Figure 5:**
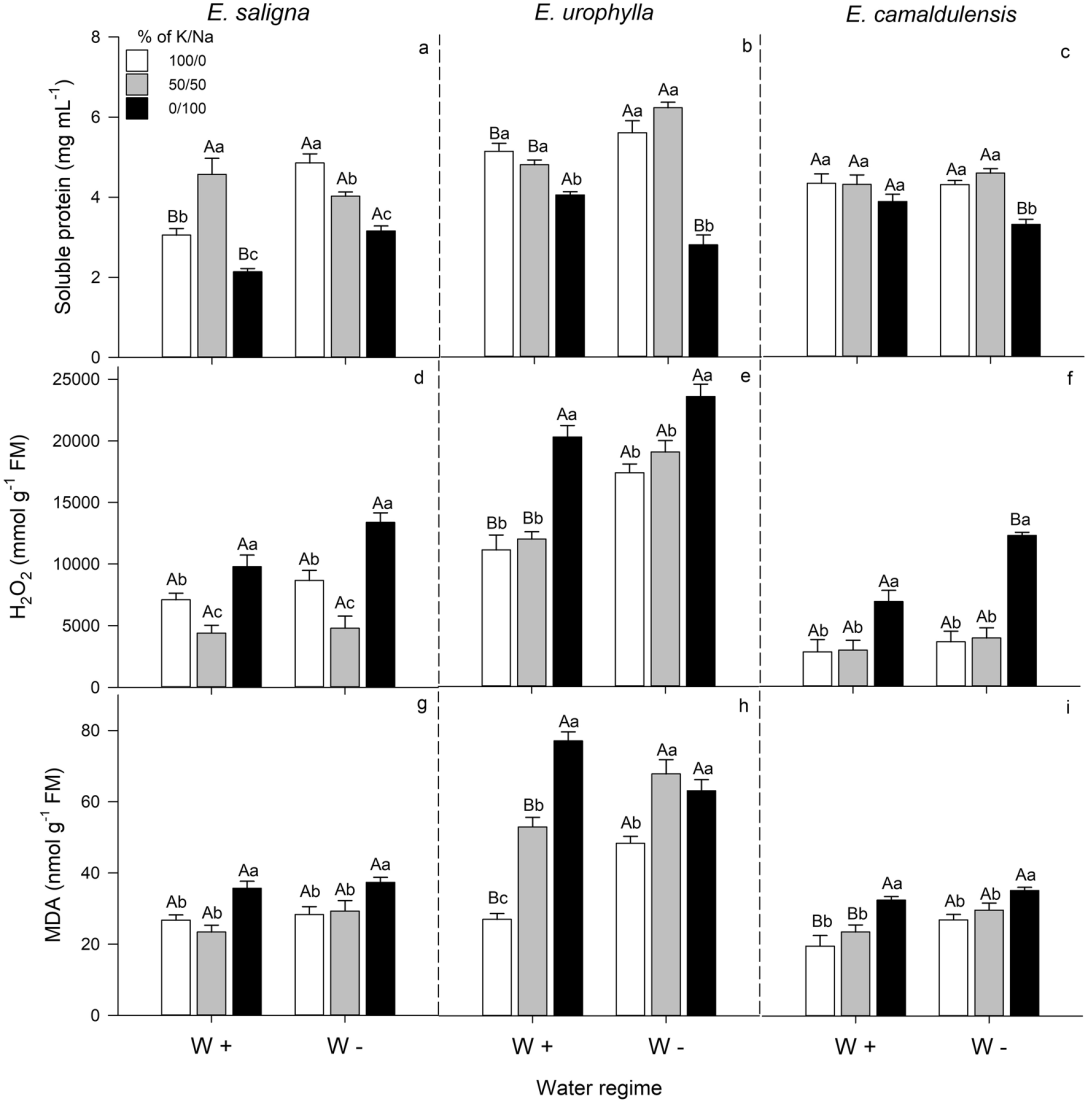
Soluble protein (**a**–**c**), hydrogen peroxide—H_2_O_2_ (**d**–**f**) and malondialdehyde—MDA (**g**–**i**) concentrations in the shoots of *E*. *saligna*, *E*. *urophylla* and *E*. *camaldulensis* seedlings under three soil levels of K/Na in well-watered (W+) and water-stressed (W−) condition. Different uppercase letters indicate difference between water conditions and different lowercase letters indicate differences between % of K replacement by Na. Vertical bars indicate standard errors between blocks (n = 4).

The well K-supply and partial K replacement by Na showed similar values in the soluble proteins, H_2_O_2_ and MDA concentration in all species, whereas the exclusive Na supply down-regulated soluble proteins, thus increasing H_2_O_2_ and MDA concentration in all species (Fig. 5).

The exclusive Na-supply decreased the soluble proteins of *E*. *saligna*, *E*. *urophylla* and *E*. *camaldulensis* relative to well K-supplied plants. The partial K replacement by Na had no effects in soluble proteins content of *E*. *urophylla* and *E. camaldulensis* under both water conditions, while increased its values in *E*. *saligna* under W+ condition, whereas reduced under W− condition, relative to well K-supplied plants. Drought stress increased the soluble protein content of *E*. *saligna* at well K-supply and exclusive Na-supply, as also for *E*. *urophylla* at well K-supply and partial K replacement by Na. However, the W− condition decreased its values in exclusive Na-supply in *E*. *urophylla* and *E*. *camaldulensis*, compared to W+ condition (Fig. 5a–c and Supplementary Table 1S).

Plants of *E*. *saligna* showed the lowest H_2_O_2_ concentration when grown under partial K replacement by Na, reducing its values by 40% under both water conditions, compared to well K-supplied plants at the same water condition. In addition, the exclusive Na-supply significantly increased the H_2_O_2_ concentration of *E*. *saligna*, *E*. *urophylla* and notably in *E*. *camaldulensis*. Drought stress showed a trend to increase the H_2_O_2_ concentration of all species: in *E*. *urophylla* by 60% at well K-supplied plants and partial K replacement by Na, and for *E*. *camaldulensis* (75%) at exclusive Na-supply, relative to W+ condition (Fig. 5d–f).

The exclusive Na-supply increased MDA of *E*. *saligna*, *E*. *urophylla* and *E. camaldulensis*, relative to well K-supplied plants at the same water condition. Moreover, the partial K replacement by Na increased the MDA of *E*. *urophylla*. Drought stress increased the MDA of *E*. *urophylla* and *E*. *camaldulensis* at well K-supplied plants and partial K replacement by Na, relative to plants under W+ condition (Fig. 5g–i).

### Stomatal density (Std), stomatal pore area and total stomatal pore area (TSPA)

Overall, the partial K replacement by Na increased the Std, while the exclusive Na-supplied plants reduced the Std for adaxial and abaxial sides, as also for TSPA under both water conditions, relative to either well K-supplied plants or the partial K replacement by Na (Table 1). Besides, no stomata were found in the adaxial side of *E*. *saligna* and *E*. *urophylla* (hypostomatic leaves, i.e. the Std was lower than 5 mm^−2^)^28^, while the leaves of *E. camaldulensis* were amphistomatic (occurred on both abaxial and adaxial sides, Supplementary Fig. 2S).

**Table 1:** Stomatal density, stomatal area and total stomatal pore area (adaxial plus abaxial surface of leaves) of *E. saligna*, *E. urophylla* and *E. camaldulensis* seedlings under three soil levels of K/Na in well-watered (W+) and water-stressed (W−) condition.

% of K/Na	Stomatal density (stomates mm^−2^)				Stomatal pore area (µm^2^)				Total stomatal pore area (10^−2^ mm^2^ mm^−2^)	
	W+		W−		W+		W−			
	Abaxial	Adaxial	Abaxial	Adaxial	Abaxial	Adaxial	Abaxial	Adaxial	W+	W−
***E. saligna***										
100/0	743.7 ± 25.7Aa	–	644.1 ± 23.4Ba	–	24.4 ± 0.2Aa	–	14.0 ± 0.4Ba	–	1.8 ± 0.1Aa	0.8 ± 0.04Ba
50/50	799.3 ± 45.9Aa	–	686.6 ± 13.8Ba	–	23.2 ± 0.8Aa	–	13.6 ± 0.8Ba	–	1.8 ± 0.1Aa	0.7 ± 0.08Ba
0/100	209.7 ± 4Bb	–	335.6 ± 10.1Ab	–	25.9 ± 0.3Aa	–	14.9 ± 0.5Ba	–	0.7 ± 0.03Ab	0.5 ± 0.01Bb
***E. urophylla***										
100/0	293.8 ± 15.7Ba	–	371.8 ± 2.4Aa	–	20.5 ± 0.2Bb	–	26.2 ± 0.5Aa	–	0.5 ± 0.03Bb	0.9 ± 0.01Aa
50/50	350.1 ± 21.9Aa	–	277.2 ± 17.2Bb	–	25.2 ± 0.4Aa	–	22.2 ± 0.6Aa	–	0.8 ± 0.04Aa	0.7 ± 0.03Aab
0/100	205.8 ± 9.4Bb	–	262.5 ± 2.4Ab	–	12.1 ± 0.3Ac	–	17.6 ± 1.0Ab	–	0.3 ± 0.01Bc	0.5 ± 0.06Ab
***E. camaldulensis***										
100/0	284.1 ± 15.7Aa	173.4 ± 4.5Aa	252.9 ± 5.67Aa	155.4 ± 3.1Aa	33.0 ± 1.8Ab	26.5 ± 3.5Bb	44.2 ± 2.6Aa	53.9 ± 2Aa	1.1 ± 0.04Bb	1.9 ± 0.06Aa
50/50	353.1 ± 21.9Aa	179.9 ± 7.8Aa	272.4 ± 4.8Ba	166.3 ± 5.0Aa	43.7 ± 2.2Aa	43.7 ± 2.3Aa	39.7 ± 0.86Aa	34.5 ± 2.1Ab	2.0 ± 0.1Aa	1.7 ± 0.1Aa
0/100	215.8 ± 20.4Ab	169.9 ± 12.3Aa	210.5 ± 2.5Ab	150.7 ± 5.4Aa	32.4 ± 1.3Ab	22.2 ± 2.1Ab	20.3 ± 0.7Bb	25.2 ± 0.9Ac	0.8 ± 0.04Ac	0.8 ± 0.04Ab

Data represent mean values and standard errors between blocks (n = 4). Different uppercase letters indicate difference between water conditions and different lowercase letters indicate differences between % of K replacement by Na according to Tukey test (p < 0.05). – Indicates that no stomata were found.

There were no significant differences between well K-supplied plants and the partial K replacement by Na in *E*. *saligna* and *E. camaldulensis* under both water conditions, and *E*. *urophylla* under W+ condition. However, the exclusive Na-supply decreased the Std of *E*. *saligna*, *E*. *urophylla* and *E. camaldulensis* compared to well K-supplied plants at the same water condition (Table 1). The adaxial Std in *E. camaldulensis* also had no significant difference among the three soil levels of K/Na. Drought stress decreased the Std of *E*. *saligna*, *E*. *urophylla* and *E. camaldulensis* in partial K replacement by Na, and of *E*. *saligna* in well K-supplied plants. Otherwise, an increase in Std was observed in *E*. *saligna* and *E*. *urophylla* with exclusive Na-supply, when compared to W+ condition.

The partial K replacement by Na increased the stomatal pore area of *E*. *urophylla* under W+ condition and *E. camaldulensis* in abaxial and adaxial side relative to well K-supplied plants (Table 1 and Supplementary Table 1S). However, the exclusive Na-supply decreased the stomatal pore area of *E*. *urophylla* and *E. camaldulensis* under W− condition in abaxial and adaxial side, compared to well K-supplied plants. Drought stress decreased the stomatal pore area of *E*. *saligna* grown under the three soil levels of K/Na and *E. camaldulensis* in abaxial side at exclusive Na-supply. Otherwise, the drought significantly increased the stomatal pore area in abaxial of *E*. *urophylla* and adaxial side of *E. camaldulensis* grown under well K-supply.

The exclusive Na-supply decreased the TSPA of *E*. *saligna*, *E*. *urophylla* and *E. camaldulensis* relative to well K-supplied plants, meanwhile the partial K replacement by Na reduced its values for *E*. *urophylla* under W− condition. Otherwise, an increase was observed for *E*. *urophylla* and *E. camaldulensis* at partial K replacement by Na under W+ condition (Table 1). Drought stress decreased the TSPA of *E*. *saligna* at well K-supply, partial K replacement by Na and exclusive Na-supply, and increased its values by 60% in *E*. *urophylla* at well K-supply and exclusive Na-supply and in *E. camaldulensis* at well K-supply, relative to W+ condition.

### Chlorophyll and flavonoids content, Na and K accumulation in plants and leaf water potential

A significant decrease was observed in chlorophyll content (Chl) and leaf water potential (Ψw) of plants under drought stress, relative to W+ condition, as also of exclusive Na-supplied plants and those under partial K replacement by Na, when compared to well K-supplied plants. The Na accumulation in plants increased by augmenting Na rates, while the K accumulation decreased by reducing K supply, according to the K/Na combination rates (Table 2 and Supplementary Table 1S).

**Table 2:** Chlorophyll content, flavonoids (Dualex units), Na and K accumulation and leaf water potential (Ψw) of *E. saligna*, *E. urophylla* and *E. camaldulensis* seedlings under three soil levels of K/Na in well-watered (W+) and water-stressed (W −) condition.

% of K/Na	Chlorophyll content		Flavonoids		Na accumulation		K accumulation		Leaf water potential (Ψw)	
	W+	W−	W+	W−	W+	W−	W+	W−	W+	W−
***E. saligna***										
100/0	18.5 ± 0.5Aa	14.6 ± 0.3Ba	3.1 ± 0.07Aa	2.9 ± 0.02Ab	0.03 ± 0.001Ab	0.03 ± 0.006Ac	0.1 ± 0.005Aa	0.1 ± 0.005Aa	− 0.80 ± 0.02Aa	− 1.06 ± 0.04Ba
50/50	11.5 ± 1Ab	9.4 ± 0.7Ab	2.5 ± 0.1Ab	2.2 ± 0.13Ac	0.06 ± 0.002Aa	0.06 ± 0.002Ab	0.05 ± 0.002Ab	0.06 ± 0.003Ab	− 0.98 ± 0.02Ab	− 1.29 ± 0.02Bb
0/100	12.0 ± 0.5Ab	7.28 ± 0.6Bb	2.9 ± 0.1Bab	3.5 ± 0.01Aa	0.05 ± 0.0006Aa	0.08 ± 0.004Aa	0.03 ± 0.002Bc	0.04 ± 0.001Ac	− 1.00 ± 0.012Ab	− 1.31 ± 0.02Bb
***E. urophylla***										
100/0	14.5 ± 0.4Aa	11.4 ± 0.5Ba	3.23 ± 0.15Aab	3.1 ± 0.06Ab	0.04 ± 0.001Ac	0.03 ± 0.003Ab	0.1 ± 0.004Aa	0.1 ± 0.006Aa	− 0.81 ± 0.01Aa	− 0.90 ± 0.03Aa
50/50	12.6 ± 0.8Aab	10.1 ± 0.2Bab	3.6 ± 0.06Aa	3.5 ± 0.07Aa	0.05 ± 0.002Bb	0.07 ± 0.001Aa	0.07 ± 0.002Ab	0.084 ± 0.002Ab	− 1.02 ± 0.07Ab	− 1.12 ± 0.05Ab
0/100	11.8 ± 0.1Ab	9.3 ± 0.06Bb	3.0 ± 0.1Ab	3.4 ± 0.04Aab	0.08 ± 0.003Aa	0.07 ± 0.002Aa	0.03 ± 0.002Ac	0.04 ± 0.002Ac	− 1.10 ± 0.01Ab	− 1.16 ± 0.05Ab
***E. camaldulensis***										
100/0	20.4 ± 0.2Aa	19.2 ± 0.4Aa	2.6 ± 0.2Aa	2.2 ± 0.1Aa	0.03 ± 0.0004Ab	0.03 ± 0.003Ab	0.1 ± 0.004Aa	0.1 ± 0.01Aa	− 0.90 ± 0.05Aa	− 1.50 ± 0.04Ba
50/50	15.7 ± 0.7Ab	12.6 ± 0.6Bb	2.6 ± 0.0Aa	2.5 ± 0.1Aa	0.06 ± 0.0008Aa	0.06 ± 0.002Aa	0.07 ± 0.0006Ab	0.07 ± 0.002Ab	− 1.10 ± 0.03Ab	− 1.77 ± 0.03Bb
0/100	17.3 ± 0.8Aab	11.2 ± 0.5Bb	2.6 ± 0.08Aa	2.3 ± 0.07Aa	0.06 ± 0.004Aa	0.06 ± 0.001Aa	0.02 ± 0.0005Bc	0.03 ± 0.0008Ac	− 1.31 ± 0.06Ab	− 1.90 ± 0.02Bb

Data represent mean values and standard errors between blocks (n = 4). Different uppercase letters indicate difference between water conditions and different lowercase letters indicate differences between % of K replacement by Na according to Tukey test (*p* < 0.05).

The partial K replacement by Na and exclusive Na-supply treatments decreased the Chl of *E*. *saligna*, *E*. *urophylla*, and *E*. *camaldulensis* relative to well K-supply (Table 2). The drought stress decreased the Chl of *E*. *saligna* at well K-supply and exclusive Na-supply, of *E*. *urophylla* at the three soil levels of K/Na, compared to W+ condition, and *E*. *camaldulensis* at partial K replacement by Na and exclusive Na-supply. The flavonoids content (Flav) decreased at partial K replacement by Na in *E*. *saligna*, whereas increased in *E*. *urophylla*, and at exclusive Na-supply in *E*. *saligna* and *E*. *urophylla* under W− condition (Table 2). Flavonoids content on *E*. *saligna* subjected to drought-induced stress increased by 22% at exclusive Na-supply, relative to plants under W+ condition.

The Na accumulation in plants augmented at partial K replacement by Na and at exclusive Na-supply relative to well K-supply. The drought stress increased the Na accumulation in plants of *E*. *saligna* at exclusive Na-supply, and of *E*. *urophylla* at partial K replacement by Na, compared to W+ condition (Table 2).

The K accumulation in plants decreased at partial K replacement by Na and at exclusive Na-supply relative to well K-supplied plants. K accumulation in *E*. *saligna* under drought-induced stress increased at partial K replacement by Na and exclusive Na-supply and of *E*. *camaldulensis* at exclusive Na-supply, compared to plants under to W+ condition (Table 2).

The partial K replacement by Na and exclusive Na-supply treatments decreased the Ψw of all eucalyptus species relative to well K-supply, at both water conditions. In addition, the drought-induced stress decreased the Ψw by 30% and 60%, respectively, of *E*. *saligna* and *E*. *camaldulensis*, at the three soil levels of K/Na compared to W+ condition (Table 2).

### Multivariate analysis: linking carbon–nitrogen isotope compositions, physiological and nutritional responses

**Figure 6:**
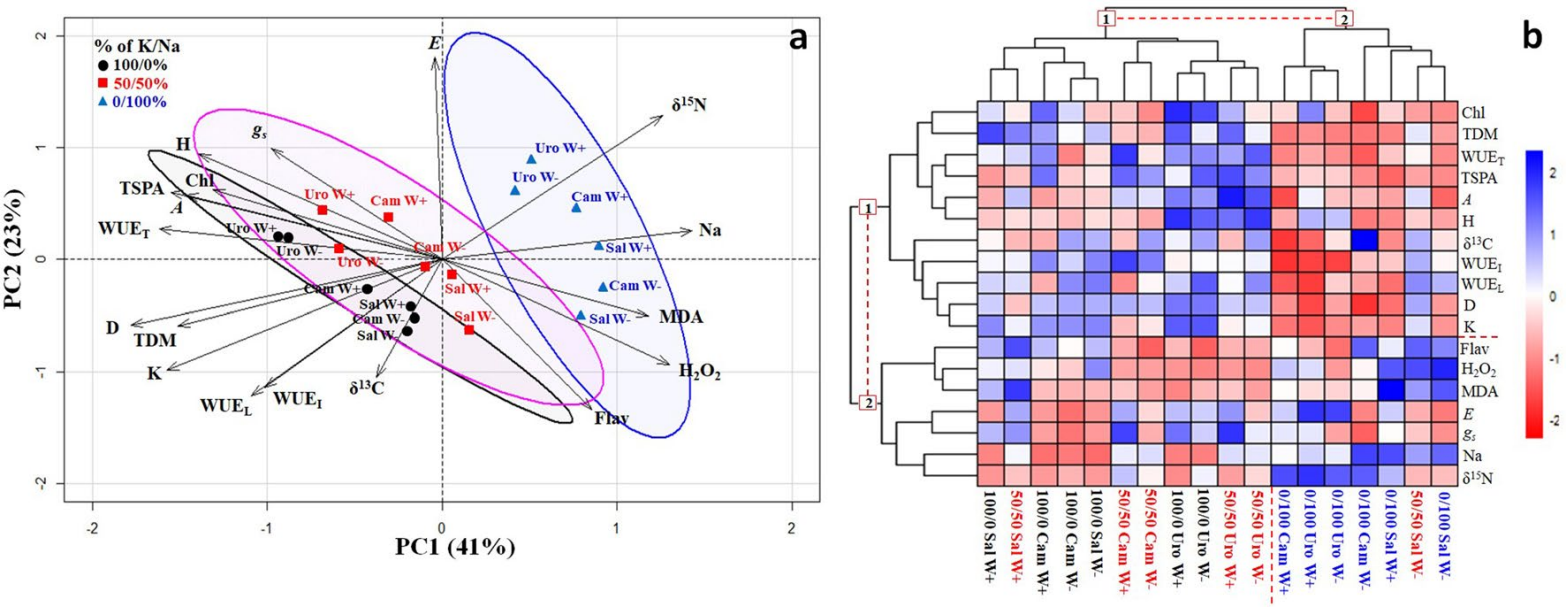
Principal component analysis—PCA with confidence ellipses (95%) (**a**) and hierarchically clustered heat map (**b**) of *E. saligna*, *E. urophylla* and *E*. *camaldulensis* seedlings under three soil levels of K/Na in well-watered (W+) and water-stressed (W−) condition. The heatmap indicates values close (blue tones) and distant (red tones). Four biological replicates were analyzed per sample (n = 4). Abbreviations: height (H), collar diameter (D), CO_2_ assimilation (*A*), stomatal conductance (*g*_s_), transpiration rate (*E*), intrinsic water use efficiency (WUE_I_), instantaneous water use efficiency (WUE_T_), long-term water use efficiency (WUE_L_), relative leaf chlorophyll content (Chl), flavonoids content (Flav), carbon and nitrogen isotope composition (δ^13^C and δ^15^N) of leaves, leaf concentration of malondialdehyde (MDA), hydrogen peroxide (H_2_O_2_), total stomatal pore area (TSPA), K and Na accumulation in plants (K and Na), total dry mass (TDM).

The PC1 and PC2 explained 41% and 23% of the total variance (Fig. 6a), respectively. The PC1 was characterized by D, TDM, TSPA, WUE_T_, K and Na accumulation and PC2 by the isotopic parameters (δ^13^C and δ^15^N), WUE_L_, WUE_I_, Flav, and *E*. Regardless of water condition, well K-supplied plants were characterized by high growth responses (H, D, TDM), WUE_T_, TSPA, *A*, Chl, K accumulation, as well as by low δ^15^N, Na accumulation, H_2_O_2_ and MDA values. Plants under partial K replacement by Na showed similar trends to the well K-supplied plants, while those under exclusive Na-supply were characterized by high δ^15^N, Na accumulation, MDA and H_2_O_2_ values.

The hierarchically clustered heat map indicated the formation of two main groups among evaluated traits and treatments (Fig. 6b). Traits were grouped into group 1, composed by growth responses (H, D and TDM), Chl, WUE, TSPA, *A*, δ^13^C, and K accumulation; and group 2, formed by Flav, H_2_O_2_, MDA, *E*, *g*_*s*_, Na and δ^15^N. The treatments were grouped into group 1, comprised by plants grown under well K-supply and partial K replacement by Na, irrespective of species and water condition, except *E. saligna* at partial K replacement by Na under W−; and group 2 was formed by plants under well K-supply, regardless species and water condition, and *E. saligna* at partial K replacement by Na under W− condition. These findings are supported by the confidence ellipse, which clearly allow the visualization of differential behavior in these two main groups of treatments, as observed by the high correlation between plants under well K-supply and partial K replacement by Na, and the higher variance in the exclusive Na-supply, irrespective of water condition and species (Fig. 6a). The δ^13^C and WUE_L_ was negatively correlated to δ^15^N in a linear relationship (*p* < 0.01, Supplementary Fig. 1S) under the three species.

## Discussion

The data obtained revealed that the partial K replacement by Na (50/50% of K/Na) up-regulated the leaf gas exchanges (*A*, *E* and *g*_*s*_), boosting WUE_L_ to maximum values in both water conditions tested, compared to the well K-supplied plants (100/0% of K/Na). The highest levels of WUE_L_ observed in plants grown under partial K replacement by Na is probably due to the ability of Na accumulation to promote not only osmotic adjustments, such as better stomatal control and lower leaf osmotic potential^9^, but also mesophyll cell enlargement, enhancing water uptake and storage^11^, thus, alleviating drought impacts in *Eucalyptus* species.

Moreover, drought decreased the Std of *E. saligna*—the drought sensitive species—grown under 100/0% and 50/50% of K/Na, which also had two-fold higher Std values than *E. urophylla* and *E. camaldulensis*, suggesting lower Std value as an effective adaptation tool for survival during dry seasons, as also observed by Frank et al.^29^. The stomatal density and its distribution at each leaf side have a clear impact on carbon physiology^30^, as observed by the positively correlation of TSPA with *A* and *g*_*s*_, being strongly driven by historic drought regime of the species^31^. In *E. saligna* and *E. urophylla*, stomata were located on the abaxial side, which is commonly observed in plants grown under mesophytic areas. However, the *E. camaldulensis* showed even Std in abaxial and adaxial side, which is common for plants grown under drought (Supplementary Fig. 2S)*.* These findings explain the greater values of *A*, *E* and *g*_*s*_ observed in *E. camaldulensis*, the drought tolerant species, compared to *E. saligna* and *E. urophylla*.

The similar plant yields observed in the treatments with K/Na rates of 100/0% and 50/50% confirm the possibility of using Na to promote CO_2_ assimilation and WUE^4^, regardless the water status. The notably positive effects of K replacement by Na in plant growth parameters of *E*. *urophylla* and *E*. *camaldulensis* than *E*. *saligna* are linked to the higher K-use efficiency^32^ in drought tolerant species, stimulating greater K uptake of plants grown under stress condition^7^. This mechanism may occur due to the higher ability to sequester Na into vacuoles, while sensitive species allocate Na mainly into cell cytosol^33^.

The stable isotope natural abundances (δ^15^N and δ^13^C) have been utilized as powerful tools to evaluate genotypic variation in WUE and integrate physiological responses to stress due to their sensitivity to environmental factors, delivering useful information about leaf gas exchange^19^, plant yield^34^ and drought-tolerant crops selection^35^. However, the WUE measurement is methodologically challenging since it can be determined via several methods, providing information on different spatial and temporal scales^36^. On a short time/leaf level scale, the WUE_I_ can be measured through the ratio of *A* by *g*_*s*_ or WUE_T_, through the ratio of *A* by *E*, producing accurate data of a specific time^36^. On the long time/whole plant scale, the WUE_L_ can also be determined by the relationship between plant dry mass yield and water consumption throughout the experimental period^37^. As observed in this study, the large increases in leaf level WUE (WUE_I_ and WUE_T_) were not reflected in significant increases in CO_2_ assimilation over the life of the plant, concealing the long-term adjustments in leaf level. This must carefully be considered when integrating these values to create more accurate and complex models for predicting WUE^31^. Thus, the WUE at the whole plant level becomes more reliable when studying gas exchanges and changes in the environmental conditions that occur during the cultivation period.

The magnitude of stomatal and/or non-stomatal limitations, that play an important role in photosynthesis, depends on the severity of the imposed stress. Under mild drought, where stomatal factors are dominant, the decline in *A* occurs as a consequence of the decrease in *g*_*s*_, and the expected trend is the increase in WUE and δ^13^C and a decrease in *C*_*i*_. However, under severe drought, non-stomatal limitations are preponderant, leading to constraints in mesophyll resistance, ribulose phosphate regeneration, rubisco activity and photochemical activity. Thus, the decrease in *A* does not occur due to a decrease in *g*_*s*_, and the expected consequence is the decrease in WUE and δ^13^C and an increase in *C*_*i*_^38,39^. As observed in our study, a highest *A*, δ^13^C and WUE was observed in plants grown under well K-supply and partial K replacement by Na, leading to a more conservative water use strategy^40^. In this context, we can assume that the stomata of plants under partial K replacement by Na were partially closed, optimizing stomatal movements to avoid water loss of *Eucalyptus* during photosynthesis. In contrast, the exclusive Na supply down-regulated stomata movements and decreased plant’s TSPA, *A*, δ^13^C and WUE while increased the *E*, due to non-stomatal factors, which may have arisen from increased affinity of RuBisCO in capturing the CO_2_ delivered by *g*_*s*_ and mesophyll conductance and isotope discrimination^39^. Regarding water condition, the drought decreased considerably the *g*_*s*_, while the WUE and δ^13^C increased, indicating that stomatal factors were responsible for the decline in *A* of *Eucalyptus* species.

The δ^13^C at 100/0% and 50/50% of K/Na increased up to 3%, 1.7% and 2% due to drought in *E*. *saligna* (drought sensitive), *E. urophylla* (moderate drought tolerance) and *E*. *camadulensis* (drought tolerance), reflecting an increase in WUE_L_ of 50%, 20% and 25%, respectively. It may be expected that even more significant increases in δ^13^C and WUE_L_ correlation would be attained for longer drought periods^41^. Thus, the δ^13^C provided a reliable estimation of the K substitution by Na effect on water status of leaves, acting as a long-term indicator of plant metabolism and integrating CO_2_ assimilation and WUE throughout the plant life. The exclusive Na supply led to the appearance of leaf chlorosis and necrosis, visual symptoms of K deficiency (Supplementary Fig. 3S), and consequently to the lower plant yield, since K is an essential element that cannot be fully replaced^42^. Meanwhile, the K-deficiency also affected plants natural profusion of ^15^N, which higher δ^15^N values indicate lower N uptake and accumulation in plant tissues, affecting plants carbon–nitrogen balance (leaf C/N ratio)^43^. As observed by the negative correlation of growth parameters and leaf gas exchange (Fig. 6a), δ^13^C and WUE_L_ (Fig. 1S) with δ^15^N, our results corroborate the utilization of δ^15^N for crop screening on N metabolism and growth conditions^44^.

The elevated ROS concentrations (H_2_O_2_), harm the cellular membranes and other cellular components, resulting in oxidative stress and cell death^45^. Potassium is responsible for stomatal movement and its deficiency may lead to stomatal closure, which causes lower light energy absorption during CO_2_ fixation and consequently intensify the electron transport chain, resulting in ROS accumulation and cell membrane oxidation^46^. As also observed for well K-supplied plants, the partial K replacement by Na maintained high values of soluble proteins, indicating higher detoxifying enzyme activities to reduce oxidative damage (lowest values of H_2_O_2_)^18^. Furthermore, the partial Na supply also relieved MDA concentration^11^ probably by entering into vacuole of stomatal cells and enabling stomatal aperture and CO_2_ fixation, decreasing therefore H_2_O_2_ synthesis^47^. Thus, partial Na supply appears to avoid oxidative stress (indicated by H_2_O_2_) and, consequently, cell membrane disruption (indicated by MDA) in plants grown under limited K supply. The Chl levels reduced with Na supply under both water conditions in all species (Table 2). These findings provide an interesting insight: partial Na supply significantly improved *A*, *g*_*s*_, and WUE_L_, besides decreasing the Chl pigment content, possibly due an efficient regulation of the available amount of light and lower ROS formation by enhanced soluble proteins.

Otherwise, the exclusive Na supply may have exceeded the capacity of H_2_O_2_ scavenging enzymes, as indicated by the low soluble protein values, depressing the photosystem activity and electron transport rate^48^ and therefore resulting in elevated concentration of H_2_O_2_ and MDA (final product of lipid peroxidation). Consequently, as typical symptom of oxidative stress, the K deficiency led to photo-oxidation of chlorophyll pigments of all *Eucalyptus* species, whose low values indicate hampered photosynthetic capacity^49^. Thus, *Eucalyptus* plants receiving less than 50% of their critical K supply could not be fully compensated by Na supply, suggesting that at least half the K requirements are necessary for functions that is poorly substituted by Na.

As mentioned, the K-deficient plants were characterized by high Na accumulation, MDA and H_2_O_2_ values. Nevertheless, the novelty was the similarity of these values with high δ^15^N and low δ^13^C and WUE_L_ (Fig. 6a). Thus, the data presented in this study confirms the potential of partial K replacement by Na in *Eucalyptus* plants with genotypes adapted to drought, such as *E.urophylla* e *E.camaldulensis*. Nonetheless, according to our hypothesis, we validate the use of natural isotopic abundance (δ^13^C and δ^15^N) as a useful sensitive indicator of WUE, stress responses and growth parameters, representing the long-term metabolism of plant life.

## Methods

### Plant material and growth conditions

The experiment was performed during the spring of 2018 with three commercial and widely cultivated *Eucalyptus* species contrasting in drought tolerance: *E*. *saligna* Sm. (sensitive); *E*. *urophylla* S.T. Blake (moderate) and *E. camaldulensis* Dehn (tolerant)^50^. Healthy and uniform 12 weeks old seedlings with 30 cm in height were obtained in a seedling nursery from Institute of Forest Research and Studies (Piracicaba city, Brazil) and transferred into pots containing 5 kg of homogenized topsoil (Oxisol soil with pH = 4.2; K = 0.3 mmol_c_ dm^−3^; and Na = 0.1 mmol_c_ dm^−3^). Seedlings were grown in a greenhouse (mean temperature of 26 °C and relative humidity of 65%) for 120 days, at the Center for Nuclear Energy in Agriculture at University of São Paulo, Piracicaba, Brazil. The soil composition was: pH = 4.0; organic matter = 15 g dm^−3^; P = 5 mg dm^−3^; S = 6 mg dm^−3^; K = 0.25 mmol_c_ dm^−3^; Ca = 1 mmol_c_ dm^−3^; Mg = 0.8 mmol_c_ dm^−3^; Al = 3 mmol_c_ dm^−3^; H + Al = 25 mmol_c_ dm^−3^; B = 0.1 mg dm^−3^; Cu = 0.7 mg dm^−3^; Fe = 30 mg dm^−3^; Mn = 0.4 mg dm^−3^; and Zn = 0.3 mg dm^−3^.

### Experimental design

The experiment was performed in randomized blocks with four replicates per treatment, in a 3 × 2 factorial design: three rates of K replacement by Na and two water conditions. Based on soil earlier trials^15^, the K replacement by Na was applied (as KCl and NaCl) in three soil levels: 100/0 (well K-supplied plants), 50/50 (partial K replacement by Na) and 0/100 (exclusive Na-supplied plants) % of K / % of Na, containing, respectively, 0/0.90, 0.44/0.44 and 0.90/0 mmol_c_ dm^−3^ of Na/mmol_c_ dm^−3^ of K, reaching the K level required for forest species development (< 1.20 mmol_c_ dm^−3^ of K)^7^.

In addition, the seedlings were fertilized with basal nutrients (nitrogen, phosphorus, calcium, magnesium, sulfur, copper, zinc, iron, boron, manganese and molybdenum), as defined by Novais et al.^51^. Sixty days after the onset of the treatments, the seedlings were watered with two different regimes: well-watered (W+) and water stressed (W −) condition. The pots were daily weighed and watered with deionized water until their respective soil relative water content reached W+ : 80% and W−: 35% respectively, accordingly to the gravimetric method^52^ to replace the evaporated and transpired water.

To analyze the influence of Na supply in extractable cations levels (K, Ca, Mg and Al), another supplementary soil incubation experiment was performed. Pots with same treatments and basal nutrients fertilization were watered daily to W+ soil relative water content and the soil was mixed monthly for 120 days and then analyzed. There were not any significant effects of Na supply (50/50 and 0/100% of K/Na) on exchangeable K, Ca, Mg and Al levels compared to the well K-supplied soil (100/0% of K/Na, data not shown).

### Leaf gas exchange

Pior to harvesting, gas exchange measures on fully expanded leaves were performed (between 9 to 11 am), with a Li-Cor 6400XT (Li-Cor, Inc., Lincoln, NE, USA). Cuvette temperature was set to 25 °C and relative humidity to 65% maintaining vapor pressure deficit in the cuvette at around 1.1 kPa. The CO_2_ concentration was kept at 380 ppm throughout all measurements. A LED array provided a photosynthetic photon flux density (PPFD) of 1,200 μmol m^−2^ s^−1^. CO_2_ assimilation (*A*), stomatal conductance (*g*_*s*_), and transpiration (*E*) were measured and averaged over 5–10 min after sample stabilization. Intrinsic water use efficiency (WUE_I_) and instantaneous water use efficiency (WUE_T_) of the leaf were determined by dividing the values of *A* by *g*_*s*_ and *A* by *E*, respectively^36^. Long-term water use efficiency (WUE_L_) was calculated by dividing the total dry mass value (belowground plus aboveground) by water use throughout the experiment (g dry mass/kg H_2_O)^37^. The leaf water potential (Ψw) was measured in the same leaves at noon (12 p.m) with a Scholander Pressure Chamber.

### Chlorophyll and flavonoids content, stomatal density and total stomatal pore area

Relative leaf chlorophyll content and flavonoids content were expressed in Dualex units, and estimated using a portable Dualex-DX4 (FORCE-A, Orsay, France). Stomatal density (Std; stomates mm^−2^) was calculated using two fully expanded leaves per plant counting both abaxial and adaxial surfaces^9^. The number of stomata was counted using ImageJ program (https://imagej.nih.gov/ij/).

The stomatal pore area (µm^2^) was considered as an ellipse (π × 0.25 × stomatal length × stomatal width)^28^, which observations were made at 1000 × magnification by scanning electron microscopy (JEOL JSM-IT300 LV, Tokyo-Japan) at 20 kV. The length and width of stomates of four randomly selected fields of view of each leaf surface were measured per plant. The total stomatal pore area (TSPA; 10^−2^ mm mm^−2^) was calculated as the product of stomatal pore area and Std.

### Growth measurements

A graduated ruler and a digital pachymeter were used to measure the growth in height (cm/plant) and collar diameter (mm/plant), respectively. Plants were harvested on the 120th day of the experiment and their leaves, stems, and roots were separated. The dry mass production of each part was calculated by drying the samples in a forced air ventilation oven at 60 °C for 72 h and then weighing them.

### Elementary and isotopic analyses

Subsequently, the plant material was ground in a Wiley type mill to quantify the K and Na concentration by nitric-perchloric digestion^53^. The accumulated K and Na (g plant^−1^) were calculated as the product of the concentration of each element in the plant part by the dry mass production of the respective tissue. To determine the leaf isotope composition (δ^13^C and δ^15^N) and the total leaf C and N, used to calculate the leaf C/N ratio, aliquots of different mass were packed into tin capsules and analyzed with an automatic nitrogen carbon analyzer (ANCA-GLS) interfaced to a continuous-flow isotope ratio mass spectrometer (Hydra 20–20, Sercon Ltd, Crewe, UK)^54^ with an analytical precision of ± 0.3‰. The isotopes composition were calculated as the following equation^20^: δ = (R_(sample)_/R_(standard)_ − 1); where R is the isotope ratio of ^13^C/^12^C or ^15^N/^14^N. The δ^13^C results were reported relative to the Vienna Pee Dee Belemnite (VPDB standard) and δ^15^N is relative to the standard atmospheric N_2_. Measurements were carried out at Isotope Ratio Mass Spectrometry Core Facility (CM-IRMS CENA) of the University of São Paulo.

### Protein extraction

The samples (0.25 g of frozen leaves) were homogenized with a mortar and pestle to 2.5 mL of 100 mmol L^−1^ phosphate buffer (pH 7.8) containing 0.04 g of PVPP and 2% Triton X-100. The homogenate compound was centrifuged at 10,000 rpm for 30 min at 4 °C, and the supernatant was stored in 0.2 mL aliquots at − 80 °C. The soluble protein concentration was determined by Bradford^55^ method using bovine serum albumin as a standard in a spectrophotometer at 595 nm (Genesys 10S UV–VIS,Thermo Fisher Scientific, Walthan, USA).

### Determination of hydrogen peroxide (H_2_O_2_) and lipid peroxidation

The leaf concentration of hydrogen peroxide (H_2_O_2_, µmol g^−1^ fresh mass^−1^) was determined according to the method described by Alexieva et al.^56^. Lipid peroxidation was estimated by the leaf concentration of malondialdehyde (MDA, nmol g^−1^ fresh mass^−1^), according to the thiobarbituric acid (TBA) test^57^. The samples (0.2 g of frozen fully-expanded leaves) were ground in 2 mL of 0.1% (w/v) trichloroacetic acid (TCA) and 0.04 g of polyvinyl polypyrrolidone (PVPP) and centrifuged at 10,000 rpm for 10 min at 4 °C. For H_2_O_2_, 0.20 mL of supernatant was added to 0.2 mL of 100 mmol L^−1^ potassium phosphate buffer (pH 7.5) and 0.8 mL of 1 mmol L^−1^ potassium iodide. The tubes remained at room temperature in the dark for 1 h in continuous dark. Readings at 390 nm were measured using a spectrophotometer (Genesys 10S UV–VIS,Thermo Fisher Scientific, Walthan, USA). Three independent replicates of each plant were used. For MDA determination, the initial procedures were the same for H_2_O_2_ measurements as described above. Following centrifugation, 0.25 mL of supernatant was added to 1 mL of 20% (w/v) TCA containing 0.5% TBA and heated in a water bath for 30 min at 95 °C. After cooling for 20 min on ice, the precipitate was removed by centrifugation at 10,000 rpm for 10 min and then, its absorbance read with a spectrophotometer (Genesys 10S UV–Vis, Thermo Fisher Scientific, Walthan, USA) at 532 and 600 nm. Three independent replicates of each plant were used. The leaf concentration of MDA was calculated as the following equation^58^:

$$C = \left[ {{\text{ABS}}\left( {535{-}600} \right) \div 155,000} \right] \times 10^{6} .$$

### Statistical procedures

The K replacement by Na and water regimes were statistically analyzed by two-way ANOVA followed by post-hoc Tukey test (*p* < 0.05) using the software SAS version 9.1 (SAS Institute Inc, 2012). The original data were standardized to be analyzed via principal components analysis (PCA), integrating the measured variables in each treatment, genotype and water condition. For the PCA, we used the treatments with Na supply for the first two principal components (PC1 and PC2) and 95% confidence ellipses to visualize the multivariate trends of Na application under W+ and W− conditions using R package (R Development Core Team, 2018). The results are indicated as mean ± standard error of four independent biological replicates and graphically visualized using SigmaPlot 11.0 (Systat Software Inc., San Jose, CA, USA).

### Research involving plants

The use of plants parts in the present study complies with international guidelines (IUCN Policy Statement on Research Involving Species at Risk of Extinction and the Convention on the Trade in Endangered Species of Wild Fauna and Flora).

## Supplementary Information

Supplementary Information.
