# Author Correction: Leaf ^13^C and ^15^N composition shedding light on easing drought stress through partial K substitution by Na in eucalyptus species

**DOI:** 10.1038/s41598-022-12821-1

**Published:** 2022-05-20

**Authors:** Nikolas Souza Mateus, Antonio Leite Florentino, Jessica Bezerra Oliveira, Elcio Ferreira Santos, Salete Aparecida Gaziola, Monica Lanzoni Rossi, Francisco Scaglia Linhares, José Albertino Bendassolli, Ricardo Antunes Azevedo, José Lavres

**Affiliations:** 1 Center for Nuclear Energy in Agriculture, University of São Paulo, Avenida Centenario, 303. CP 96, Piracicaba, CEP 13416-000 Brazil; 2 College of Agriculture Luiz de Queiroz, University of São Paulo, Piracicaba, 13418-900 Brazil

Correction to: *Scientific Reports* 10.1038/s41598-021-99710-1, published online 11 October 2021

The Acknowledgements section in the original version of this Article was incomplete.

“This work was supported financially in part by the National Council for Scientific and Technological Development (CNPq, Project # 137864/2017-5) and the agreement of the Coordination of Superior Level Staff Improvement (CAPES) and the São Paulo Research Foundation (FAPESP, Project # 2017/24410-4). JL and RAA thanks the National Council for Scientific and Technological Development from Brazil (“Conselho Nacional de Desenvolvimento Científico e Tecnológico” – CNPq) for the research fellowship (Grants # 303718/2020-0 and 303749/2016-4, respectively).”

now reads:

“This work was supported financially in part by the National Council for Scientific and Technological Development (CNPq, Project # 137864/2017-5) and the agreement of the Coordination of Superior Level Staff Improvement (CAPES) and the São Paulo Research Foundation (FAPESP, Project # 2017/24410-4 and # 2019/16168-4). JL and RAA thanks the National Council for Scientific and Technological Development from Brazil (“Conselho Nacional de Desenvolvimento Científico e Tecnológico” – CNPq) for the research fellowship (Grants # 303718/2020-0 and 303749/2016-4, respectively).”

The original Article has been corrected.

