# Supplementary material for: Leaf 13C and 15N composition shedding light on easing drought stress through partial K substitution by Na in eucalyptus species
Source: Sci Rep. 2021 Oct 11;11:20158. doi: 10.1038/s41598-021-99710-1 (PMC8505639; doi:10.1038/s41598-021-99710-1)
Supplement: Supplementary file 1 — Supplementary Information. [file 41598_2021_99710_MOESM1_ESM.pdf]

Supplementary material

Leaf ^{13}C and ^{15}N composition shedding light on easing drought stress through partial K substitution by Na in eucalyptus species

Nikolas Souza Mateus*[1], Antonio Leite Florentino[2], Jessica Bezerra Oliveira[1], Elcio Ferreira Santos[1], Salete Aparecida Gaziola[2], Monica Lanzoni Rossi[1], Francisco Scaglia Linhares[1], José Albertino Bendassolli[1], Ricardo Antunes Azevedo[2], Jose Lavres*[1]

Table 1S- Percentage of increase (in blue color) or decrease (in red color) of each significant analyze in *E. saligna*, *E. urophylla* and *E. camaldulensis* grown under partial K replacement by Na (50/50% of K/Na) and exclusive Na-supply (0/100% of K/Na) relative to the well K-supply (100/0% of K/Na), in well-watered (W +) and water-stressed (W -) condition. – indicate no significant differences.

	Partial K replacement by Na (50/50% of K/Na)						Exclusive Na supply (0/100% of K/Na)					
	E. saligna		E. urophylla		E. camaldulensis		E. saligna		E. urophylla		E. camaldulensis	
	W+	W-	W+	W-	W+	W-	W+	W-	W+	W-	W+	W-
Height	-	-	-	-	-	-	-13%	-20%	-18%	-17%	-23%	-19%
Collar Diameter	-	-	-	-	-	-	-20%	-35%	-25%	-22%	-35%	-25%
Total dry matter	-30%	-20%	-	-	-	-	-45%	-35%	-50%	-35%	-50%	-35%
A	77%	36%	70%	30%	23%	20%	-45%	-	-	-45%	-30%	-40%
E	210%	195%	210%	30%	-	-	165%	-	165%	-	55%	55%
g_s	280%	100%	-	45%	-	-	115%	-	-30%	-	-35%	-60%
WUE_I	-	-	-	-	-	-	-55%	-20%	-15%	-25%	-40%	-45%
WUE_T	-	-	-	-	-	-	-55%	-25%	-20%	-30%	-45%	-55%
WUE_L	-	-25%	-	-	-	-	-25%	-40%	-40%	-25%	-50%	-40%
$\delta^{13}C$	-	-	-	-	-	-	-2%	3%	-2%	-2%	-3%	-2%
$\delta^{15}N$	-	-	-	-	-	-	85%	65%	55%	35%	100%	35%
C/N ratio	-27%	-	22%	-	18%	-	-60%	-30%	-20%	-20%	-15%	-20%
Soluble Protein	50%	-20%	-	-	-	-	-30%	-35%	-20%	-50%		-25%
H_2O_2	-40%	-40%	-	-	-	-	35%	55%	80%	35%	135%	240%
MDA	-	-	95%	40%	-	-	30%	35%	180%	35%	65%	30%
Std_{AB}	-	-	-	-25%	-	-	-70%	-45%	-30%	-30%	-25%	-15%
Stomatal pore area	-	-	23%	-	45%	-		-	-40%	-32%	-	-53%
TSPA	-	-	37%	-36%	70%	-	-60%	-40%	-46%	-48%	-25%	-55%
Chl	-35%	-43%	-17%	-20%	-19%	-38%	-35%	-43%	-17%	-20%	-19%	-38%
Flav	-19%	-22%	13%	11%	-	-	-	22%	-	7%	-	-
Na accum	60%	62%	46%	94%	56%	53%	54%	120%	106%	117%	60%	52%
K accum	-50%	-40%	-30%	-25%	-45%	-40%	-70%	-60%	-67%	-65%	-85%	-70%

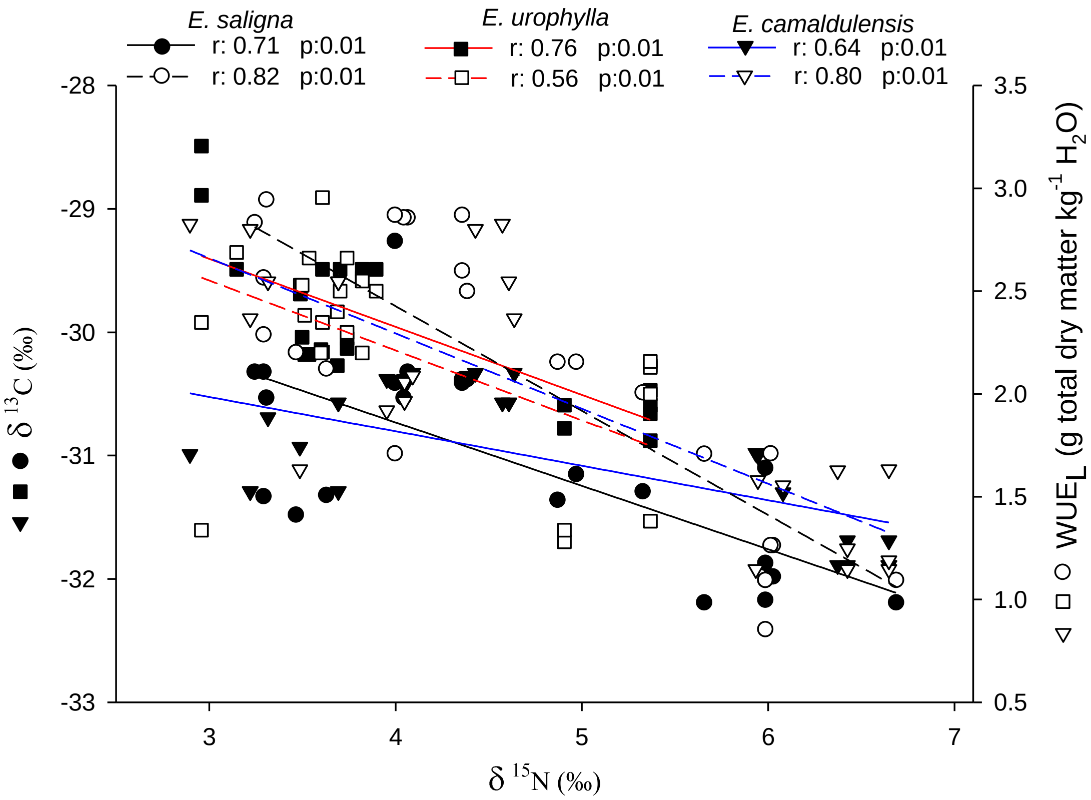

Figure 1S- Relationship among $\delta^{13}C$, $\delta^{15}N$ and long-term water use efficiency (WUE_L) of *E. saligna*, *E. urophylla* and *E. camaldulensis* seedlings under three soil levels of K substitution by Na (100/0%, 50/50%, and 0/100% of K/Na) in well-watered and water-stressed condition.

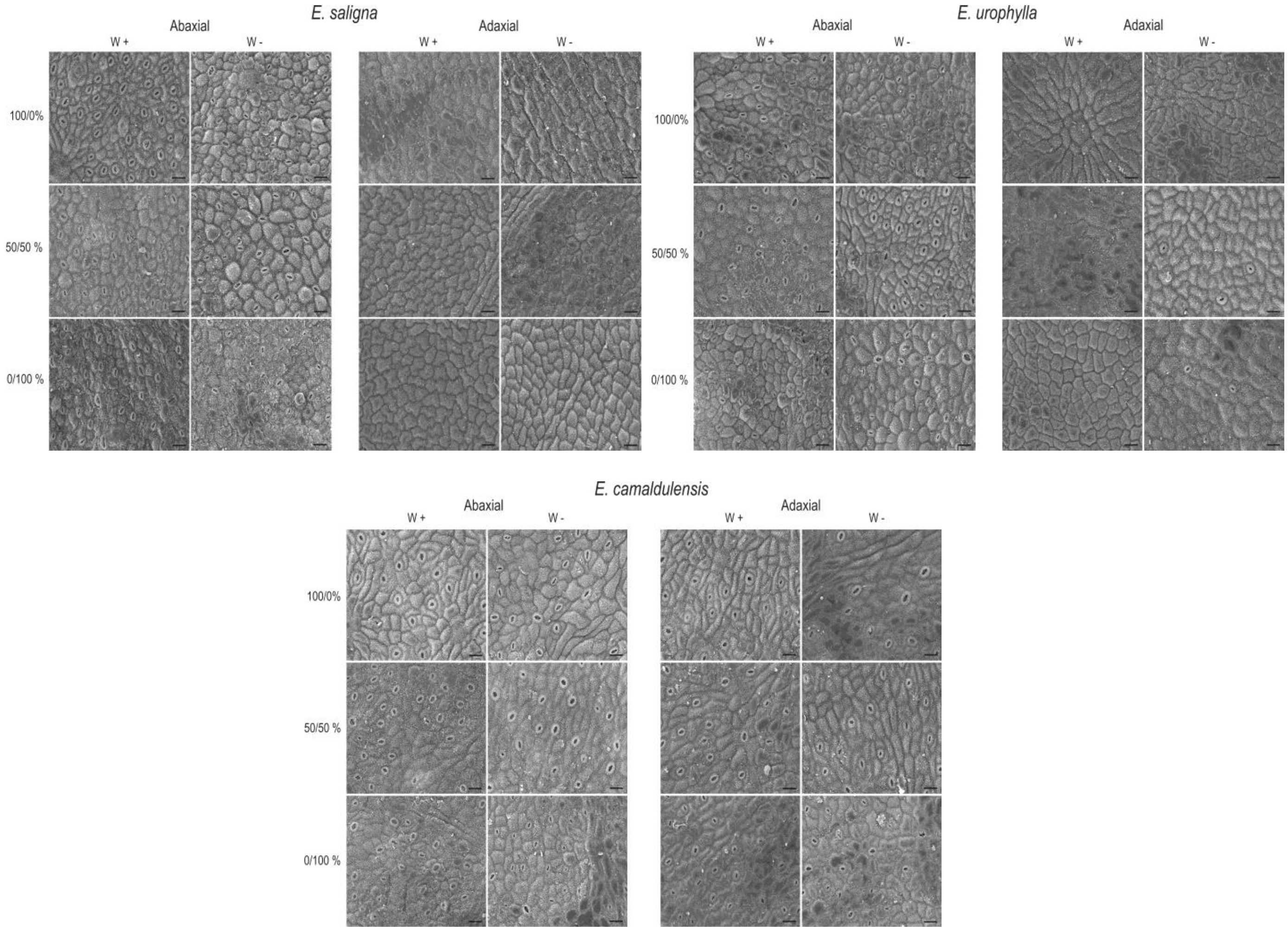

Figure 2S- Micrograph augmented 100x of scanning electron microscopy in abaxial and adaxial side of well K-supplied plants (100/0% of K/Na), partial K replacement by Na (50/50% of K/Na) and exclusive Na-supplied plants (0/100% of K/Na) of *E. saligna*, *E. urophylla* and *E. camaldulensis* seedlings in well-watered (W +) and water-stressed (W -) condition.

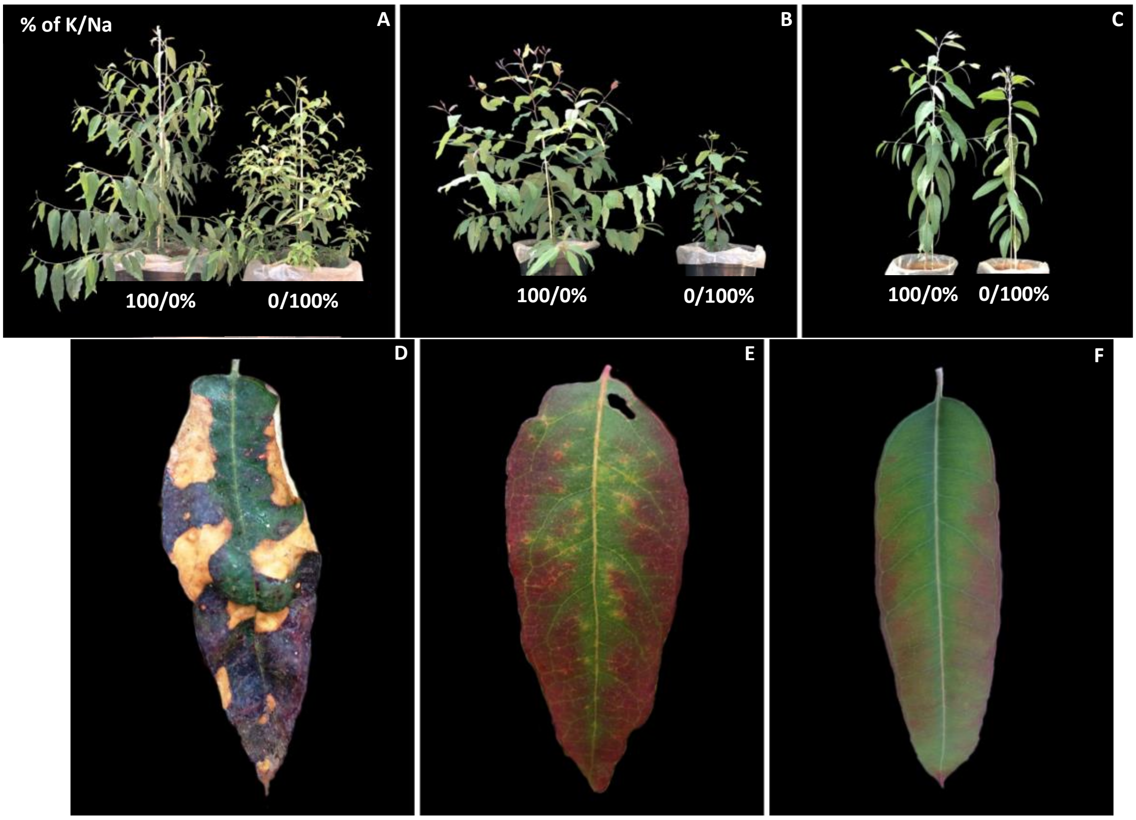

Figure 3S- Well K-supplied plants (100/0% of K/Na) and exclusive Na-supplied plants (0/100% of K/Na) with visual symptom of K deficiency in mature leaves of *E. saligna* (A and D), *E. urophylla* (B and E) and *E. camaldulensis* (C and F) grown under well-watered condition, 90 days after the onset of treatments.
